# Maintained mitochondrial integrity without oxygen in the anoxia-tolerant crucian carp

**DOI:** 10.1242/jeb.247409

**Published:** 2024-07-01

**Authors:** Mark A. Scott, Cathrine E. Fagernes, Göran E. Nilsson, Kåre-Olav Stensløkken

**Affiliations:** ^1^ Section for Physiology and Cell Biology, Department of Biosciences, University of Oslo, 0361 Oslo, Norway; ^2^ Division of Physiology, Department of Molecular Medicine, Institute of Basic Medical Sciences, University of Oslo, 0372 Oslo, Norway

**Keywords:** Mitochondria, Heart, Metabolism

## Abstract

Very few vertebrates survive without oxygen (anoxia) for more than a few minutes. Crucian carp (*Carassius carassius*) are one example, surviving months of anoxia at low temperatures, and we hypothesised that they maintain mitochondrial membrane potential and function. Isolated crucian carp cardiomyocytes indeed maintained mitochondrial membrane potential after blocking complex IV of the electron transport system with cyanide, while those of anoxia-intolerant trout depolarised. When complexes I–III were inhibited, crucian carp mitochondria depolarised, indicating that these complexes need to function during anoxia. Mitochondrial membrane potential depended on reversal of ATP synthase in chemical anoxia, as blocking with cyanide combined with oligomycin to inhibit ATP synthase led to depolarisation. ATP synthase activity was reduced in the heart after 1 week of anoxia in crucian carp, together with a downregulation of ATP synthase subunit gene expression. However, the morphology of cardiac mitochondria was not affected by 1 week of anoxia, even with a large increase in mitofusin 2 mRNA expression. Cardiac citrate synthase activity was not affected by anoxia, while cytochrome *c* oxidase activity was increased. We show how mitochondria respond to anoxia. A mechanistic understanding of how mitochondrial function can be maintained in anoxia may provide new perspectives to reduce mitochondrial damage in anoxia-sensitive organisms.

## Teaser

**Summary:** Analysis of cardiac and red skeletal muscle of crucian carp exposed to anoxia shows both mitochondrial function and morphology are maintained, possibly through the continued activity of complexes I–III in the electron transport system.

## INTRODUCTION

While most vertebrates die rapidly in anoxia, there are a few exceptions (Fago, 2022). The crucian carp (*Carassius carassius*) is one of the best established models of vertebrate anoxia tolerance, being able to survive and maintain physical activity during anoxia about 1000 times longer than most vertebrates (Nilsson and Lutz, 2004). This is accompanied by a unique capacity to maintain cardiac output without oxygen (Stecyk et al., 2004). Long-term cardiac function and survival without oxygen also implies viable mitochondria. Still, how crucian carp mitochondria respond to anoxia is less known, and it has not been examined whether they maintain mitochondrial membrane potential (ψ_m_), or allow their mitochondria to depolarise – an event that initiates cell death in other vertebrates.

The evolution of anoxia tolerance allows the crucian carp to survive in ice-covered ponds and lakes that become anoxic during the winter. The crucian carp then relies on ATP from glycolysis, where the anaerobic end-product is ethanol, produced in part by a mutated variant of Enzyme 1 in the pyruvate dehydrogenase complex (Fagernes et al., 2017).

Oxygen is essential as a final electron acceptor in the electron transport system (ETS). The flow of electrons through complexes I–IV pumps protons into the intermembrane space of the mitochondria, which flows back through the ATP synthase that converts ADP to ATP. In mammals, prolonged absence of oxygen will lead to a disturbance of electron flow, a fall in ATP, loss of ψ_m_ and initiation of cell death cascades (Chouchani et al., 2014). Complex II of the ETS will reverse and lead to an accumulation of succinate (Chouchani et al., 2014). Moreover, accumulation of NADH and lactate lead to a highly reduced state and inhibition of glycolysis, which further reduces ATP production (Fago, 2022). In mammals, substrate-level phosphorylation can prolong cell and mitochondrial survival for a short time (Kiss et al., 2014), but not for weeks as is the case for the crucian carp.

We hypothesised that the crucian carp maintains cardiac mitochondrial morphology and membrane potential in anoxia, thereby preventing mitochondria from becoming inducers of cell death. In several of the experiments, we made comparative measurements on cardiomyocyte mitochondria from rainbow trout (*Oncorhynchus mykiss*), a commonly used representative of the vast majority of vertebrates that cannot survive long-term anoxia. As the crucian carp is an anoxia survivor, we also hypothesised that there are changes in the metabolic profile of the heart, with an upregulation of glycolytic intermediates.

Our findings show that mitochondrial membrane potential is maintained for at least 33 h in chemical anoxia, but is dependent on complexes I–III. Moreover, we show that mitochondrial integrity is maintained during prolonged anoxia (1 week).

## MATERIALS AND METHODS

The laboratory work was performed at the Department of Biosciences at the University of Oslo. All experiments were approved and conducted in accordance with the regulation on the use of experimental animals for scientific purposes in Norway (National Animal Research Authority of Norway, Mattilsynet, FOTS # 12007). Fish [*Carassius carassius* (Linnaeus 1758) and *Oncorhynchus mykiss* (Walbaum 1792)] were held in tanks supplied with aerated dechlorinated tap water (8–12°C), fed daily and maintained under a 12 h:12 h light:dark regime. Details on experimental procedures and other methods are provided in Supplementary Materials and Methods and briefly described below. The study complied with the ARRIVE guidelines.

### Isolation and evaluation of cardiomyocytes

All chemicals were purchased from Sigma-Aldrich unless otherwise stated. Cardiomyocytes were isolated based on a modified protocol (Vornanen, 1997), where hearts initially were gravity perfused with isolation media (in mmol l^−1^: 100 NaCl, 10 KCl, 1.2 KH_2_PO_4_, 4 MgSO_4_, 50 taurine, 20 glucose and 10 Hepes, titrated to pH 6.9 with KOH) for 15 min to remove any excess blood cells. During enzymatic digestion (18 min for carp, 6 min for trout), 1.3 mg ml^−1^ collagenase type 1A, 1 mg ml^−1^ BSA and 1 mg ml^−1^ trypsin were added to the perfusate. The partly digested tissue was mechanically disrupted by pipetting before cardiomyocytes were sedimented for 20 min on glass slides and rinsed in isolation media prior to the start of the experiments.

For viability experiments, isolated cardiomyocytes were incubated in isolation media with sodium cyanide, iodoacetate (0.1, 1 and 10 mmol l^−1^) or a combination of the two for 1 h before cells were rinsed and stained with Hoechst (# 33342, 1 µmol l^−1^), Mitotracker (M22426, 5 nmol l^−1^) and propidium iodide (40 µmol l^−1^, P3566) (all ThermoFisher) for 60 min; 0.1, 1 and 10 mmol l^−1^ NaCl were used as vehicle controls. Cells were rinsed twice in PBS before being fixed in 2% paraformaldehyde and mounted with a gelatin/glycerol medium before adherence to coverslips.

For live cell experiments, cardiomyocytes were stained with 12 µmol l^−1^ Hoechst and 30 nmol l^−1^ tetramethylrhodamine methyl ester perchlorate (TMRM, ThermoFisher) in isolation media for 30 min at 10°C (temperature maintained throughout the experiment) in glass bottom culture dishes (MatTek, P35G-0-14-C). In the next media change, the following chemicals was added to individual chambers (in μmol l^−1^); 5, 0.5, 0.05, 0.005 carbonyl cyanide *m*-chlorophrenylhydrazone (CCCP), 1000 NaCN (1000 NaCl as control), 0.03 oligomycin A, 5 rotenone and 5 antimycin A. The concentration of CN^−^ in isolation media over time was measured semi-quantitatively with colorimetric strips using the Mquant Cyanide test (Merck, 1.10044.0001) according to the manufacturer's protocol. NaCN in isolation media was kept at 10°C in a 6-well plate and measured at 0, 3, 24 and 33 h. The concentration of CN^−^ decreased slightly with time, but was not below 500 µmol l^−1^ after 33 h. The intensity of TMRM was evaluated in the ScanR fluorescence microscopy platform (Olympus IX81, inverted) equipped with a Hamamatsu C8484-05G camera. The microscope was programmed to take 18 images per well at the same location in each well. To avoid bleaching and TMRM-induced reactive oxygen species (ROS) production, each well was only imaged once, together with a control. Cardiomyocytes from each fish was therefore seeded into 6–10 parallel culture dishes and kept in the dark at 10°C before being imaged after the different exposures. The TMRM intensity from the experimental groups was compared with vehicle control groups and expressed as percentage difference from the control.

### Anoxia exposure, tissue sampling and enzyme activity

Crucian carp of both sexes (*n*=110 for all experiments, 22±6 g) were placed in opaque 25 l containers and allowed to acclimate for 24 h with aerated water flow (8–12°C, same as holding temperature). Anoxia was achieved by bubbling nitrogen into a supply column in addition to the container, making the oxygen level fall below 0.01 mg O_2_ l^−1^ (detection limit of the oxygen electrode) within 6 h. A galvanometric oxygen electrode (Oxi 340i: WTW) was used to verify the concentration of oxygen. Normoxic individuals were held in a parallel container that was bubbled with air. Reoxygenation was achieved by subjecting crucian carp exposed to 1 week of anoxia to normoxic conditions for 1 week before sampling. Animals were killed by a sharp blow to the head, and tissues were immediately sampled and snap frozen in liquid nitrogen within 1 min of confirmed death, and stored at −80°C until further analysis. Fish were not fed during exposures.

The activity of citrate synthase (CS) and of cytochrome *c* oxidase (COX) was measured spectrophotometrically according to the manufacturer’s protocol and earlier work in non-mammalian species (Dalziel et al., 2012; Duerr and Podrabsky, 2010). In brief, samples were ground into a powder in a liquid nitrogen-cooled mortar and pestle, followed by sonication of 15 mg tissue in ice-cold buffer containing 20 mmol l^−1^ Hepes, 5 mmol l^−1^ EDTA and 0.1% Triton X-100, pH 7.0. A spectrophotometer (Perkin Elmer) was used at room temperature (20°C) and the data were analysed with UV Kinlab software (Perkin Elmer). ATP synthase activity was measured using an ATP regenerating system described by Rosing et al. (1975) and used in non-mammalian species (Duerr and Podrabsky, 2010). Final cuvette concentrations for each assay were as follows: CS – 0.1 mmol l^−1^ 5,5′-dithiobis-(2-nitrobenzoic acid) (DTNB), 0.3 mmol l^−1^ acetyl CoA and 0.1 mmol l^−1^ oxaloacetate in 10 mmol l^−1^ Tris, pH 7; COX – 10 µmol l^−1^ reduced cytochrome *c* in 10 mmol l^−1^ Tris, pH 7; and ATP synthase – 2 mmol l^−1^ ATP, 1.5 mmol l^−1^ phosphoenolpyruvate, 0.17 mmol l^−1^ NADH, 6 U pyruvate kinase, 12 U lactate dehydrogenase and 0.6 µmol l^−1^ oligomycin in 33 mmol l^−1^ Tris-acetate, 83 mmol l^−1^ sucrose and 10 mmol l^−1^ MgCl_2_, pH 7.2. All assays were optimised to ensure that substrates were not limiting. Samples were analysed at 412 nm (CS), 550 nm (COX) and 340 nm (ATP synthase). Bradford's reagent (B9616) was used to assess total protein content.

### Electron microscopy

Details on tissue preparation and average mitochondrial volume estimation are given in Supplementary Materials and Methods. In short, red skeletal muscle and heart were pinned down during fixation with 2.5% glutaraldehyde in PBS. After 2 h, a 1 mm^3^ block was cut and stored in fixative until analysis. Mitochondrial volume was estimated blindly with a stereological method using Cavalieri's Principle (West, 2012) from six randomly selected locations from each animal (*n*=5). In short, a grid was placed on top of each mitochondrial electron microscope image and the number of times the grid intersect over mitochondria was counted. At each location, five serial sections with a distance of 0.74 µm were used. The five sections were counted for the number of disappearing profiles. A volume for each mitochondria was then estimated using the formulas provided in Supplementary Materials and Methods. The number of mitochondria was calculated from the same images taken with a Philips CM200 transmission electron microscope.

### Cloning, sequencing and qPCR

The genomic sequence of crucian carp is not known; therefore, consensus sequences were made based on available zebrafish and mammalian species and aligned using GeneDoc (version 2.7) and Clustal X (version 2.0.12) (Fagernes et al., 2017). Primers were designed using Primer3 (Rozen and Skaletsky, 2000), aiming at highly conserved regions for the sequences aligned (Table S3). Tissue samples were spiked with an external standard gene (mw2060), a protocol designed specifically for severe physiological challenges when standard housekeeping genes might change (Ellefsen et al., 2008). mRNA was extracted using TRIzol (Invitrogen) and target genes were cloned, sequenced and blasted to confirm correct sequence. For each sample, two cDNA syntheses were performed, both with duplicated qPCR reactions according to our previously published work (Fagernes et al., 2017).

### Metabolomics

Tissue were sampled and transported on dry ice to the Human Metabolome Technologies facility in Yamagata, Japan. In brief, samples from normoxic and 7 day anoxic crucian carp hearts (*n*=6) were diluted in acetonitrile (50%) and internal standards were added, before being homogenised, filtered and concentrated. Results were obtained using capillary electrophoresis-time of flight mass spectroscopy (CE-TOFMS) and triple quadruple mass spectroscopy (CE-QqQMS) analysis. Further details are found in Supplementary Materials and Methods.

### Statistics

Statistical analysis was performed using GraphPad Prism (version 9.3.1). Values in the text and figures are reported as means±s.d. unless otherwise stated. Two-way ANOVA with Holm–Šidák's multiple comparison tests were used to evaluate differences between species and blockade of ETS components. Kruskal–Wallis test with a Dunn's multiple comparison tests were used to test differences in enzyme activity and gene expression. A principal component analysis was performed by SampleStat version 3.14 (in-house software) for the metabolomics data and *P*-values were computed using Welch's *t*-test.

## RESULTS

### Maintained mitochondrial membrane potential in crucian carp cardiomyocytes after blocking complex IV with sodium cyanide

**Fig. 1.**
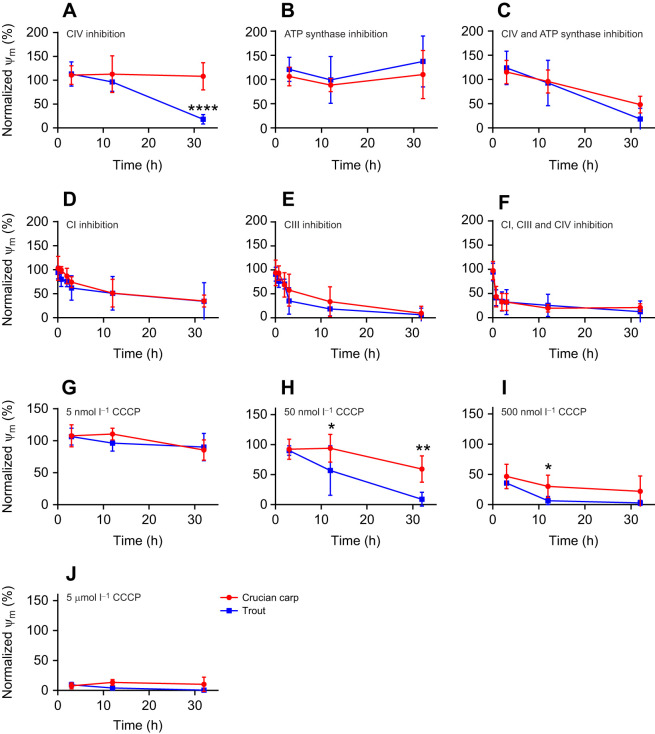
**Normalised mitochondrial membrane potential (ψ_m_) of isolated cardiomyocytes from rainbow trout and crucian carp exposed to blockers of the electron transport system (ETS) and the uncoupler CCCP.** Data (means±s.d., *n*=6 for each species) were normalised to control cell ψ_m_ measured at each point. (A) Complex IV inhibition with 1 mmol l^−1^ sodium cyanide. (B) ATP synthase inhibition with 30 nmol l^−1^ oligomycin A. (C) Complex IV and ATP synthase inhibition with a combination of 1 mmol l^−1^ sodium cyanide and 30 nmol l^−1^ oligomycin. (D) Complex I inhibition with 5 µmol l^−1^ rotenone. (E) Complex III inhibition with 5 µmol l^−1^ antimycin A. (F) Complex I, III and IV inhibition with combined 5 µmol l^−1^ rotenone, 5 µmol l^−1^ antimycin A and 1 mmol l^−1^ sodium cyanide treatment. (G–J) Uncoupling of the proton gradient with increasing concentrations of CCCP: 5 nmol l^−1^ (G), 50 nmol l^−1^ (H), 500 nmol l^−1^ (I) and 5 μmol l^−1^ (J). Statistical differences were investigated with a two-way ANOVA, with Holm–Šidák's *post hoc* test (*P<0.05, **P<0.01, ****P<0.0001).

First, we examined whether the ψ_m_ is maintained even when oxygen cannot be used as final electron acceptor at complex IV. To investigate this, isolated cardiomyocytes from crucian carp and trout were exposed to sodium cyanide (a blocker of oxygen binding to complex IV) *in vitro*, and ψ_m_ was measured with fluorescence microscopy after loading the mitochondria with TMRM. Crucian carp cardiomyocyte mitochondria maintained ψ_m_ even after 33 h of cyanide exposure, while those of trout had depolarised by this time (Fig. 1A). Blocking the ATP synthase in normoxia with oligomycin had no significant effect on normoxic cardiomyocyte ψ_m_ (Fig. 1B). However, blocking both complex IV and ATP synthase led to a loss of membrane potential in crucian carp mitochondria (Fig. 1C), suggesting that ATP synthase is initially working in reverse as an ATPase that pumps out H^+^ in order to help maintain ψ_m_. When inhibiting complex I and III, three additional time points were investigated (15 min, 45 min and 2 h), to reveal acute differences between the species. Interestingly, inhibiting complex I with rotenone or complex III with antimycin made both species lose ψ_m_, revealing that the first part of the ETS needs to be functional to allow a maintained ψ_m_ even in anoxia-tolerant crucian carp (Fig. 1D,E). As a result, inhibiting all complexes in the ETS effectively depolarised ψ_m_ both in trout and in crucian carp (Fig. 1F). It should be mentioned that one or more of the inhibitors used may be broken down or evaporate during the 33 h long experiment, even at a relatively low temperature. Some decrease was seen for cyanide, but it was still above 500 µmol l^−1^ after 33 h. What argues against a significant loss of inhibition is that many of the effects seen developed from 12 to 33 h (Fig. 1), including the progression to full depolarisation of cyanide-exposed trout mitochondria.

To investigate whether the crucian carp could resist proton leakage, the proton gradient was challenged with increasing doses of the uncoupler CCCP. While both crucian carp and trout could maintain ψ_m_ at the lowest dose (5 nmol l^−1^; Fig. 1G), only crucian carp were able to do this when exposed to 50 nmol l^−1^ CCCP (Fig. 1H). Also, at 500 nmol l^−1^ CCCP, crucian carp maintained a higher ψ_m_ than trout (Fig. 1I). ψ_m_ was depolarised by 5 µmol l^−1^ CCCP in both species (Fig. 1J).

**Fig. 2.**
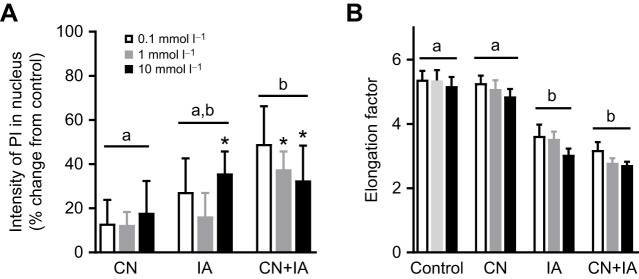
**Crucian carp cardiomyocyte viability and length/width relationship in response to inhibition of oxidative phosphorylation and glycolysis.** (A) Cell viability, assessed as the intensity of propidium iodide (PI) in nuclei, in isolated cardiomyocytes exposed to increasing doses (0.1, 1 or 10 mmol l^−1^) of the complex IV inhibitor sodium cyanide (CN), the glycolysis inhibitor iodoacetate (IA) or CN+IA in combination. (B) Elongation factor of cardiomyocytes following complex IV (CN treatment) and/or glycolysis (IA treatment) inhibition. A decrease in elongation factor indicates hypercontracted and less functional cardiomyocytes. Data are means±s.e.m. (*n*=5). Statistical differences were investigated with a two-way ANOVA, with Holm–Šidák's *post hoc* test. Different letters denote a significant difference between treatments (*P*<0.05). Asterisks denote a significant difference from controls for a given concentration within a treatment (*P<0.05).

Without oxidative phosphorylation, cells are largely dependent on glycolysis to produce sufficient ATP. We therefore investigated crucian carp cardiomyocyte viability in response to three doses of sodium cyanide and in combination with the glycolysis inhibitor iodoacetate. As expected, cyanide did not increase cell death, while the highest dose of iodoacetate alone decreased viability (Fig. 2A). Blocking both oxidative phosphorylation and glycolysis significantly increased cell death even at the lowest doses (Fig. 2A). Similarly, the elongation factor (length/width relationship) of the carp cardiomyocytes was decreased after treatment with iodoacetate alone, or in combination with sodium cyanide (Fig. 2B). A decrease in elongation factor indicates hyper-contracture and less functional cardiomyocytes. There was no difference in elongation factor between control and cyanide-treated cardiomyocytes, or between cardiomyocytes treated with iodoacetate alone or in combination with cyanide (Fig. 2B).

### Decreased ATP synthase activity and reduced expression of the ATP synthase subunit C in crucian carp hearts after 7 days of anoxia

Having established that crucian carp cardiomyocytes are dependent on reversed ATP synthase activity to maintain ψ_m_ in the acute phase of chemical anoxia (Fig. 1C), we investigated the activity of this enzyme after 1 week of *in vivo* anoxia exposure. We appreciate the exposure time difference between the *in vitro* (33 h) and *in vivo* experiments. However, we did not want to extend the *in vitro* protocol as we would expect this to introduce confounding factors such as bacterial growth in the media and loss of inhibitor activity. Conversely, 33 h of anoxia *in vivo* would be a rather short exposure time for crucian carp, which survive several weeks of anoxia at this temperature. In our group, we also routinely use 7 days of anoxia exposure to be able to compare *in vivo* data between studies. Also, in defence of the time difference, it could be argued that the *in vitro* situation for isolated cardiomyocytes in a dish is in any case radically different from being a working cardiomyocyte in a working heart in a whole organism.

**Fig. 3.**
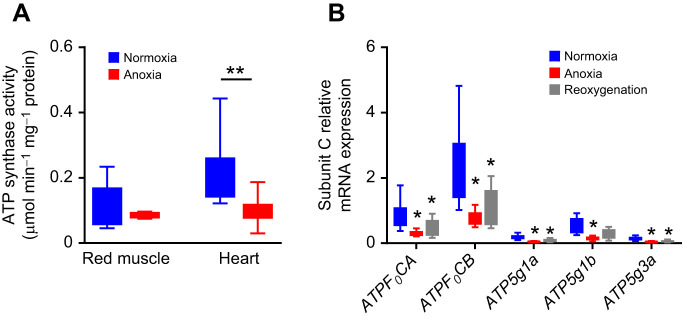
**ATP-synthase activity and subunit C expression in the heart and red skeletal muscle of crucian carp exposed to anoxia and reoxygenation.** (A) ATP synthase activity in the heart and red skeletal muscle from crucian carp exposed to anoxia (<0.01 mg O_2_ l^−1^, *n*=8) for 1 week compared with normoxic controls (*n*=8). (B) mRNA expression levels of five paralogues of subunit C (rotation ring of F_1_ in the ATP synthase) in hearts from crucian carp exposed to normoxia (*n*=10), 1 week of anoxia (*n*=9), and 1 week of anoxia followed by 1 week of normoxia (reoxygenation, *n*=10). mRNA expression level was normalised to an external standard (mw2060). Data are presented as boxplots (25–75 percentiles) with 5–95 percentile error bars. A Mann–Whitney test was used for ATP synthase activity, while a Kruskal–Wallis test with Dunn's *post hoc* test was used for mRNA expression level (*P<0.05, **P<0.01).

We found reduced ATP synthase activity in the crucian carp heart but not in red skeletal muscle (Fig. 3A). To investigate whether ATP synthase could be suppressed at the mRNA level, we cloned, sequenced and performed qPCR on selected members of the F_1_ subunit of the ATP synthase: assembly factor for the alpha and beta subunit (ATPAF2 and ATPAF1, respectively) and the gamma subunit (ATP5C1). The level of ATP5C1 mRNA expression was reduced in anoxic hearts but not in red skeletal muscle (Table S1). For the F_0_ subunit, we investigated subunit C and found mRNA expression of five paralogues (*ATPF_0_CA*, *ATPF_0_CB*, *ATP5g1a*, *ATP5g1b* and *ATP5g3a*) of which all were downregulated in anoxic hearts (Fig. 3B), but not in red skeletal muscle (Table S1). Subunit C of ATP synthase has been suggested to be the pore forming unit of mitochondrial permeability transition pore (MPTP) that mediates cell death (Carraro et al., 2020). For the F_0_ e-subunit, we found four paralogues of which two showed reduced mRNA expression levels in anoxic hearts but not in red skeletal muscle (Table S1). An important regulator of mammalian ATP synthase activity is inhibitory factor 1 (IF-1). However, mRNA expression levels of the two paralogues of IF-1 that we found (*IfA* and *IfB*) were not significantly altered in the heart or red skeletal muscle of crucian carp during anoxia (Table S1).

In addition to ATP synthase subunit C, other candidates or interacting partners of the MPTP have been suggested, and we investigated their mRNA expression in heart tissue and red skeletal muscle. The adenine nucleotide translocators (ANT1, 2 and 3) have been suggested as pore candidates (Carraro et al., 2020). ANT2 had the highest overall mRNA expression in crucian carp tissue (Table S1) and expression of both of its paralogues (*ANT2.1* and *ANT2.2*) decreased in anoxic hearts (Table S1). *ANT2.1* expression also increased in reoxygenated muscle (Table S1). Expression of the two paralogues of ANT3 also decreased in the anoxic heart (Table S1). The voltage-dependent anion channel (VDAC) was previously suggested as a MPTP candidate (Winquist and Gribkoff, 2020). *VDAC2* had the highest expression in both heart and red skeletal muscle but we found no change in expression of *VDAC1*, *VADC2* or *VADC3* in response to anoxia (Table S1). Linked to VDAC in MPTP pathophysiology is hexokinase (Winquist and Gribkoff, 2020), the first enzyme in glycolysis. Of the hexokinases, subtype 1 (*Hex1*) had the highest expression (Table S1). *Hex2.2* and *Hex4.1* expression levels increased in anoxic hearts compared with control, while all the subtypes increased in reoxygenated hearts (Table S1). Also, in red skeletal muscle, expression levels of *Hex1* and *Hex2.2* increased after reoxygenation (Table S1). Cyclophilin D is not considered as a structural part of MPTP but increased levels favour pore opening (Carraro et al., 2020). In the heart, we found an increased expression level of cyclophilin D (*Cyc D*) during reoxygenation, while it was reduced in red skeletal muscle (Table S1). Furthermore, it has been suggested by others that members of the mitochondrial solute carrier protein (SLC25) family, including phosphate carriers (PiC) and citrate carrier (Cits) could be regulators of the MPTP (Carraro et al., 2020). However, we found no change in expression of *PiC 1*, *PiC 2* and *Cits2* in the tissues (Table S1). Interestingly, we found a reduction in expression of the transcription factors *tFAM1* and *tFAM2* in anoxic red skeletal muscle, while no change was found in the heart (Table S1). This could indicate that mitochondrial replication was indeed affected by anoxia.

### Increased mitochondrial volume in anoxic carp red skeletal muscle but not in the heart

**Fig. 4.**
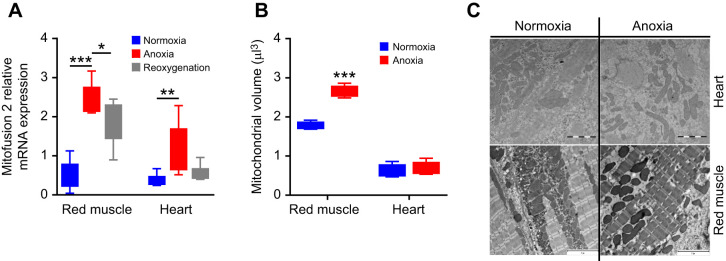
**Mitofusion 2 mRNA expression and mitochondrial volume in the heart and red skeletal muscle of crucian carp exposed to anoxia and reoxygenation.** (A) Mitofusin 2 mRNA expression level in crucian carp exposed to anoxia (<0.01 mg O_2_ l^−1^) for 1 week (*n*=8) or 1 week of anoxia followed by 1 week of normoxia (reoxygenation, *n*=6) compared with normoxic fish (*n*=6). mRNA expression level was normalised to an external standard (mw2060). (B) Estimation of mitochondrial volume using stereology on electron microscopy images of red skeletal muscle and heart from normoxic and anoxic crucian carp (*n*=5). Data are presented as boxplots (25–75 percentiles) with 5–95 percentile error bars. Kruskal–Wallis test with Dunn's *post hoc* test was used for gene expression, while a Mann–Whitney test was used for mitochondrial volume (*P<0.05, **P<0.01, ***P<0.001). (C) Representative electron microscopy image from the stereological evaluation of mitochondrial volume. Scale bars: 2 µm (heart) and 5 µm (red muscle).

Given the maintained mitochondrial function in carp cardiomyocytes without oxygen, we wanted to investigate whether the morphology of mitochondria differed between normoxia and anoxia in the crucian carp. Here, we made comparisons of samples from heart and red skeletal muscle from the same individuals. Mitofusins are membrane tethering proteins controlling fusion of the outer mitochondrial membrane and intracellular mitochondrial transport (Dorn, 2020). After 1 week of anoxia, a 3- and 6-fold increase in mRNA expression of mitofusin 2 was found in the heart and red skeletal muscle when compared with normoxia, respectively (Fig. 4A). For mitofusin 1, two paralogues where found (*Mit1.1* and *Mit1.2*), and one of these (*Mit1.1*) showed increased mRNA expression in the heart during anoxia compared with normoxia (Table S1).

We counted the number of mitochondria in the electron microscope images that were used for mitochondrial volume estimation. We found no change in the mitochondrial count between normoxia and anoxia in the tissues, but the mitochondrial volume of red skeletal muscle increased by 48% during anoxia compared with normoxia (Fig. 4B). Representative electron micrographs of normoxic and anoxic crucian carp red skeletal and cardiac muscles from the stereological analysis are shown in Fig. 4C. Note also the absence of glycogen in the anoxic tissue, visible as black dots in normoxia but not seen after a week of anoxia (Fig. 4). Because of differences in mitochondrial size between heart and skeletal muscle, the magnification on the volume estimation is different, but this was accounted for in the calculation of volume.

### Increased COX activity in the heart but not in red skeletal muscle

**Fig. 5.**
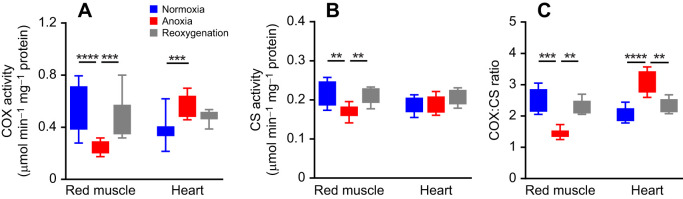
**Cytochrome *c* oxidase (COX) and citrate synthase (CS) activity in the heart and red skeletal muscle of crucian carp exposed to anoxia and reoxygenation.** (A) COX and (B) CS activity in crucian carp after 1 week of anoxia (*n*=12) or 1 week of anoxia followed by 1 week of normoxia (reoxygenation, *n*=11) compared with normoxic fish (*n*=10). (C) Ratio of COX to CS activity. Data are presented as boxplots (25–75 percentiles) with 5–95 percentile error bars. A Kruskal–Wallis test with Dunn's *post hoc* test was used for analysis (**P<0.01, ***P<0.001, ****P<0.0001).

As the abundance and morphology of mitochondria during anoxia appeared unaffected, we investigated COX activity in red skeletal muscle and cardiac tissues from crucian carp exposed to normoxia and 1 week of anoxia. We found an increase in COX activity in the heart and a reduction in the red skeletal muscle of the anoxia group (Fig. 5A). In the red skeletal muscle, CS activity was reduced during anoxia, while we found no change in the heart (Fig. 5B). Consequently, 1 week of anoxia induced an increase in the COX/CS ratio in hearts, while it led to a reduction in the red skeletal muscle (Fig. 5C).

### The importance of redox state and fumarate as a possible electron acceptor in carp mitochondria during anoxia

**Fig. 6.**
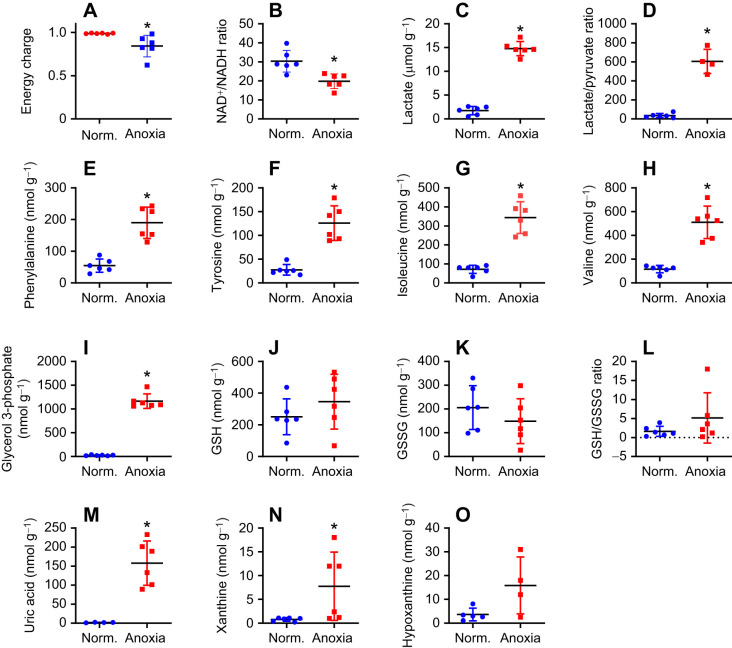
**Metabolomics analysis of anoxic crucian carp hearts.** Energy charge (A), NAD^+^/NADH ratio (B), lactate (C), lactate/pyruvate ratio (D), phenylalanine (E), tyrosine (F), isoleucine (G), valine (H), glycerol 3-phosphate (I), reduced (GSH; J) and oxidised (GGSG; K) glutathione and GSH/GSSG ratio (L), uric acid (M), xanthine (N) and hypoxanthine (O) levels (means±s.d., *n*=6) for crucian carp exposed to normoxia or to anoxia (<0.01 mg O_2_ l^−1^) for 1 week. Statistical analysis was done with a Welch corrected *t*-test (*P<0.05). Some metabolites were for unknown reasons not detected in some samples, resulting in fewer data points.

**Fig. 7.**
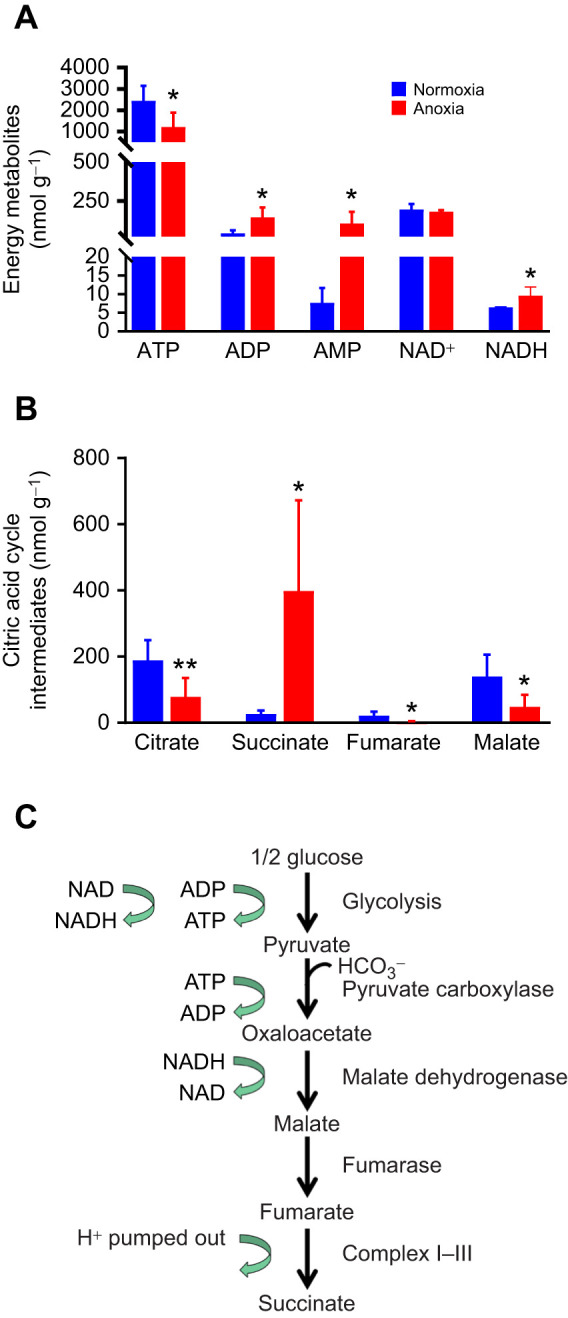
**Energy metabolites and citric acid cycle intermediates in the heart of crucian carp exposed to anoxia.** Levels of energy metabolites (A) and intermediates from the citric acid cycle (B) from normoxic or 1 week anoxic hearts. Data are means±s.d. (*n*=6 for each). Statistical analysis was done with a Welch corrected *t*-test (*P<0.05, **P<0.01). (C) A possible metabolic route to fumarate. All the absolute concentrations of the 116 metabolites investigated in this experiment can be found in Table S2.

Our *in vitro* experiments revealed that crucian carp cardiomyocytes need a sustained function of complexes I–III in order to maintain their ψ_m_, while ψ_m_ can be maintained during chemical anoxia (induced by cyanide) where only complex IV is blocked. To further investigate the underlying mechanisms, we performed a metabolomics analysis of more than 100 metabolites, comparing cardiac tissue exposed to 1 week of anoxia with normoxic tissue. As expected, the adenylate energy charge in the heart was relatively well maintained during anoxia, still being 0.8 (Fig. 6A). There was a significant reduction in the ATP level following anoxia, while ADP and AMP levels increased, possibly reflecting a new steady state during suppressed metabolism (Fig. 7A). To avoid a highly reduced state and thereby an inhibition of glycolysis, a high NAD^+^/NADH ratio needs to be maintained. Although the NAD^+^/NADH ratio was reduced in anoxia, the absolute level of NAD^+^ was maintained (Figs 6B and 7A). Moreover, there was an expected increase in lactate (Fig. 6C) and increased lactate/pyruvate ratio in anoxic hearts compared with normoxic hearts (Fig. 6D).

Levels of citric acid cycle intermediates, including fumarate, were decreased in anoxic hearts compared with normoxic hearts, except for succinate, which increased by 14-fold (from 27.0±9.3 to 398±249 nmol g^−1^) (Fig. 7B). Concomitantly, cardiac levels of the fumarate-related amino acids phenylalanine and tyrosine increased during anoxia compared with normoxia (Fig. 6E,F), which was also found for two succinyl CoA-related amino acids, isoleucine and valine (Fig. 6G,H). These findings indicate a putative role of fumarate acting as an electron acceptor while producing succinate. This mechanism was recently shown in mammals (Spinelli et al., 2021). Notably, the succinate level increase exceeded the decrease of fumarate, suggesting that fumarate is continuously produced during anoxia in cardiac tissue. An overview of all the metabolites measured, comparing normoxic and anoxic tissue, is provided in Table S2. A possible metabolic route to fumarate is given in Fig. 7C.

Another important mechanism for maintaining cytoplasmic redox state is the dihydroxyacetone phosphate (DHAP) to glycerol 3-phosphate conversion, which is coupled to the NADH to NAD^+^ conversion. We found a 44-fold increase in glycerol 3-phosphate in anoxic hearts compared with normoxic hearts (from 26.3±8.9 to 1165± 139 nmol g^−1^; Fig. 6I). Moreover, cardiac levels of uric acid, xanthine and hypoxanthine increased by 100-fold, 10-fold and 5-fold, respectively, in anoxia compared with normoxia (Fig. 6M–O).

## DISCUSSION

We show here that crucian carp cardiomyocyte mitochondria maintain ψ_m_ even if oxygen binding to complex IV is blocked by cyanide, a treatment termed chemical anoxia, for at least 30 h. Our findings point to mechanisms by which the maintenance of ψ_m_ in anoxia is achieved. Firstly, blocking ATP synthase (complex V) with oligomycin A during chemical anoxia resulted in a loss of ψ_m_, suggesting that during acute anoxia this enzyme is initially running in reverse, working as a proton pumping ATPase. This has also been described in the anoxia-tolerant turtle with suggestions of the involvement of IF-1 (Hawrysh and Buck, 2019). However, we found no transcriptional regulation of IF-1 in crucian carp hearts. Secondly, blocking complexes I and III resulted in ψ_m_ depolarisation, suggesting that the first parts of the ETS remain functional during anoxia and are needed to ensure proton pumping in order to maintain ψ_m_. The cardiac metabolomics data showed a large increase in anoxic succinate levels, similar to previous reported levels in different crucian carp tissues (Dahl et al., 2021). It is therefore possible that complex I (NADH-ubiquinone oxidoreductase) remains active in anoxic hearts and works together with complex II to use fumarate as an electron acceptor to produce succinate while simultaneously pumping protons into the mitochondrial intermembrane space. Fumarate as an electron acceptor, supporting partial complex I activity without oxygen, has been described in mammals (Spinelli et al., 2021). Complex I activity might prevent mitochondrial depolarisation, with its detrimental consequences. This mechanism may be particularly important as complex V appears to be suppressed during anoxia, as suggested by our finding of decreased ATP synthase activity and decreased expression of ATP synthase subunits, particularly in the heart, after 1 week of anoxia exposure *in vivo*. In mammals during complex I inhibition, substrate-level phosphorylation can provide ATP for short periods of time through the α-ketoglutarate dehydrogenase complex while NAD^+^ is supplied through diaphorases (Kiss et al., 2014; Ravasz et al., 2018). Our results also indicate that complex III is necessary to maintain mitochondrial membrane potential. We have no good explanation for this, but crucian carp have an evolutionary advantage with two genome duplications (Fagernes et al., 2017), which could allow the fish to evolve special molecular mitochondrial mechanism to tolerate anoxia.

In isolated cells, we found that reversed ATP synthase was necessary for maintaining mitochondrial membrane potential, by acting as a proton pump. As this mechanism consumes ATP, it is arguably unfavourable for prolonged anoxic survival, when anaerobic glycolysis is the only major ATP-producing pathway (St-Pierre et al., 2000). This could be the reason for the suppression of ATP synthase displayed by the intact anoxic crucian carp heart. Similar mechanisms are also suggested for the turtle heart (Bundgaard et al., 2019a). The reduction in ATP synthase activity is much larger in the turtle heart than in the crucian carp (Gomez and Richards, 2018). It is possible that ATP synthase activity is also necessary for maintaining the membrane potential after a week in anoxia *in vivo*, but *in vivo* measurement of mitochondrial membrane potential is technically difficult. However, our results suggest a transcriptional suppression in prolonged anoxia, as we found major reductions in the levels of mRNA for several of the ATP synthase subunits and a 50% reduction in the cardiac ATP synthase activity.

These observations point to the possibility that complex I performs a task in maintaining ψ_m_. If fumarate serves as an electron acceptor, how is it made available, and how is the accumulation of succinate handled? We did see a fall in fumarate levels in the heart during anoxia, but it was not completely depleted and the fall in fumarate on a mole-to-mole basis (decreasing from 22 to 3.6 µmol l^−1^) was just a fraction of the accumulation seen in succinate (rising from 27 to 399 µmol l^−1^) during anoxia. There are some alternatives for how fumarate could be continuously supplied in crucian carp during anoxia (Dahl et al., 2021; Lefevre and Nilsson, 2023). In one pathway, oxaloacetate is produced from glucose via pyruvate in transamination reactions that include glutamate pyruvate transaminase and glutamate oxaloacetate transaminase (Vanwaarde et al., 1982). Oxaloacetate can then be converted to malate and subsequently to fumarate by reversal of a part of the tricarboxylic acid (TCA) cycle. This pathway includes the consumption of aspartate and the production of alanine, and our results show that cardiac aspartate levels do indeed fall during 1 week of anoxia while there is an increase in alanine. In a second pathway, aspartate can be converted to fumarate through the purine nucleotide cycle. Both these pathways appear to be active in anoxic crucian carp (Dahl et al., 2021). We also see a third possibility which involves production of oxaloacetate from pyruvate by pyruvate carboxylase, an enzyme that is known to occur in the genus *Carassius* (Van den Thillart and Smit, 1984). Oxaloacetate can then be converted to fumarate as in the first pathway, and the overall reaction involves no net consumption of ATP or NAD^+^ (Fig. 7C).

From this, there are clearly possibilities for crucian carp tissues to produce fumarate from glucose and aspartate during anoxia, particularly as crucian carp are known to have the largest glycogen stores of any vertebrate (Nilsson, 1990). These glycogen stores are used for ATP production during anoxia by anaerobic glycolysis with ethanol as the major glycolytic end-product in the genus *Carassius* (Fagernes et al., 2017). As expected, in the electron microscopy images, we saw abundant glycogen in normoxic tissue, which was absent in anoxic tissue of both heart and red muscle. We would like to point out the possibility that the glycogen stores can be used for the continuous production of fumarate and eventually succinate during anoxia to allow for proton pumping by the first parts of the ETS. However, this leaves the challenge of succinate processing. Even if succinate levels are raised a lot in relative terms (15-fold), the concentration of succinate described in cardiac tissue after 1 week of anoxia is still modest (0.4 mmol l^−1^), but 4 times higher than in anoxic turtle heart and 10 times lower than an ischaemic mouse heart (Bundgaard et al., 2019a). If this is all the succinate that was produced, it can hardly have contributed to proton pumping over a long time. The possibility remains that it is stored elsewhere in the body or even excreted.

As the major role for mitochondria is to produce ATP when oxygen is present, one could hypothesise a general reduction in mitochondria when the crucian carp is facing prolonged anoxia. However, we saw no reduction in the number of mitochondria in the images we quantified after 1 week of anoxia in either the heart or the skeletal muscle. These results comply with previous findings in anoxia-tolerant turtle hearts, where there was no reduction in mitochondrial volume, number, protein content or enzyme activity (Bundgaard et al., 2019b). This suggests that for anoxia-tolerant vertebrates, it is important to maintain the integrity and number of mitochondria in anoxia. Interestingly, crucian carp showed a large increase in mitochondrial volume in red skeletal muscle after 1 week of anoxia, together with a 6-fold increase in mitofusin 2 mRNA expression in red skeletal muscle, compared with normoxia. Mitofusins are membrane tethering proteins that control fusion of the outer mitochondrial membrane (Dorn, 2020). Data also suggest that mitofusin 2 is important for intracellular mitochondrial transport and also as an anchoring point for the mitophagic protein PINK1 (Dorn, 2020). Loss of mitofusin 2 leads to small fragmented mitochondria of bad quality (Dorn, 2020). It is possible that the up-regulation of mitofusin 2 in both the heart and skeletal muscle of anoxic crucian carp is involved in maintaining healthy mitochondria during periods without oxygen (Fagernes et al., 2017).

CS is the first step in the citric acid cycle and often used to estimate mitochondrial content. For the heart, we found no changes in CS activity, which, together with the unchanged volume, supports maintained mitochondrial content. In red skeletal muscle, we saw a small but significant reduction in CS activity after 1 week of anoxia, contrasting the increase in volume. It is important to note that the red skeletal muscle mitochondria have another important role in anoxia, as the mutated paralogue of pyruvate dehydrogenase is located here, where it is responsible for ethanol production (Fagernes et al., 2017). We found a large reduction in COX activity in skeletal muscle. COX is the fourth complex in the mitochondrial ETS and delivers electrons to oxygen while producing water. Again, the main role of muscle mitochondria in anoxia is not oxidative phosphorylation but conversion of lactate produced in other organs into ethanol. This could also explain the maintained cardiac output in anoxia (Stecyk et al., 2004). Surprisingly, we found an increase in anoxic COX activity in the heart, although the cyanide experiments suggested that the cardiomyocytes do not depend on this enzyme to survive and maintain ψ_m_. These results might indicate that the entire ETS is preserved and ready to be utilised when oxygen returns.

An important factor in the mammalian intolerance to anoxia is the occurrence of the mitochondrial permeability transition, or the opening of a large pore in the mitochondrial membrane (MPTP) leading to the release of molecules up to 1500 Da (Hausenloy et al., 2020). The molecular identity of the pore is unknown, but one hypothesis is that it consists of parts of subunit C of ATP synthase (Hausenloy et al., 2020). As crucian carp can survive months of anoxia, the mitochondrial permeability transition does not take place, and some mechanism must exist to prevent formation of the MPTP. The anoxic crucian carp heart displayed a large reduction in mRNA expression levels of several isoforms of ATP synthase subunit C, which could partly explain prevention of the formation of MPTP. Over the years, several mitochondrial proteins have been suggested to play a regulatory role in MPTP, including ANT, VDAC, hexokinase and PiC. As there is less ATP and ADP to transport across the mitochondrial membranes, ANT mRNA expression levels could be expected to decrease. ANT has been suggested as a candidate for the pore together with cyclophilin D (Karch et al., 2019). We found no reduction of cyclophilin D mRNA expression during anoxia in either the heart or skeletal muscle, but decreases in ANT2, and ANT3 mRNA expression in the heart. It is possible that the mitochondria may form both large and small pores (Hausenloy et al., 2020) and that the combined downregulation of both ANT and the ATP synthase subunit C in the crucian carp plays a role in preventing MPTP opening.

Finally, our findings of increased expression of most of the hexokinase paralogues is most likely linked to increased glycolysis in anoxia, as hexokinase is the first regulatory step in this pathway. As these are gene expression data only, we cannot conclude about the physiological importance of such findings.

### Conclusion

This study provides information on how cardiac and red skeletal muscle cells of crucian carp maintain both mitochondrial function and morphology during events of complete anoxia. Maintained ψ_m_ in crucian carp hearts appears to be supported by continued activity of complexes I–III in the ETS, possibly using fumarate as the electron acceptor. In addition to the maintained ψ_m_, downregulation of pore-forming proteins possibly decreases MPTP formation. From a biomedical perspective, studying anoxia-tolerant mitochondria could provide perspectives in the search for measures to counteract mitochondrial damage during conditions such as ischaemia and reperfusion.

## Supplementary Material

10.1242/jexbio.247409_sup1 Supplementary information
