# Supplementary material for: Maintained mitochondrial integrity without oxygen in the anoxia-tolerant crucian carp
Source: J Exp Biol. 2024 Jul 1;227(20):jeb247409. doi: 10.1242/jeb.247409 (PMC11418198; doi:10.1242/jeb.247409)
Supplement: Supplementary information [file jexbio-227-247409-s1.pdf]

Supplementary Material and Methods

Animals

Crucian carp (*Carassius carassius*) were wild-caught from the Tjernsrud pond, Oslo municipality. They were kept on a 12h:12h L:D regime in flow through tanks (~50 fish per 250 L) supplied with aerated and dechlorinated Oslo tap water (8-12ºC). Fish were fed a maintenance diet daily with commercial carp food (Tetra Pond, Tetra, Melle, Germany). Wild Brown trout (*Salmo trutta*) were supplied by Oslomarkas Fiskeadministrasjon in Sørkedalen, Oslo municipality; fed pellets from Skretting (Spirit Ørret 300 – 4.5 mm), and housed in the same facilities as the crucian carp, 10 fish per tank. All animals were given at least one week for acclimatization to holding conditions and fasted for 24 h before any experiments were conducted. The animal experiments was approved and performed according to the National Animal Research Authority of Norway (permit nr 12007), and all methods involving research animals were performed in accordance with relevant guidelines and regulations.

Fluorescence Microscopy

Fluorescence microscopy was used for quantifying changes in mmp ($\Delta\Psi_M$) and cell death. The protocol used was similar to that of Bliksøen *et al*. (Bliksoen et al., 2016). The ScanR fluorescence microscopy platform (Olympus IX81, inverted) is a automated system that incorporates the detection, imaging, and counting of nuclei and cells (as well as the fluorescent intensity of stained mitochondria within cells). The system is objective as the same locations are imaged within each well, independent of intensities of the stained cells. Automated image analysis software CellR (Olympus) with an edge detection algorithm was used for analysis. For the fixed-cell IA experiments images were taken at eight automated locations in each well. For each fish and treatment cellular morphology and fluorescent intensity was averaged from readings for 139±93 cells. Controls and treatments were performed in duplicate. For the live-cell TMRM experiments the readings at each time point were made on independent wells so as to avoid repeated measures on TMRM-loaded cells. Studies have shown that the photoexcitation of TMRM produces ROS (Brady et al., 2004). It was important to limit ROS production in this study because free radical production could accelerate the depolarization of $\Delta\Psi_M$. Additionally, TMRM was selected over TMRE and Rhod123 because TMRM is less likely to inhibit the ETC (Perry et al., 2011).

Electron Microscopy

Ultra thin sections (80 nm) were cut with a diamond knife (Diatome, Switzerland) on a Leica Ultracut UCT (Leica Microsystems, Germany) ultramicrotome and placed on 75 mesh pre-coated copper grids (Electron Microscopy Sciences). A Philips CM200 transmission electron microscope was used to image sections of heart and RM tissue at a magnification of 2750X. Volume was estimated using Cavalieri's Principle, which states that "the volume of an arbitrary-shaped object can be estimated in

Journal of Experimental Biology • Supplementary information

an unbiased manner from the product of the distance between planes and the sum of the areas on systematic parallel sections through the object" (Garcia et al., 2007). In other words, it is used to overcome the challenge of describing an inner three dimensional form based on the analysis of structural slices containing only two dimensional information. For reviews, illustrations, and details of calculations see (Mandarim-de-Lacerda, 2003; Mayhew, 1992; West, 2012). Before analysis, micrographs were assigned a random number in order to blind the researcher from knowing which were normoxic controls and which were anoxic treatments. The 'point-counting' method was used to profile the area of mitochondria on the first section. The method involves placing a grid atop the micrograph and the number of times the grid intersects over mitochondria is counted. The 5 sections were counted for the number of disappearing profiles. These are the number of instances in a given section where mitochondria are no longer visible that were present in the preceding section. From these values, the estimated mitochondrial volume was calculated:

$$Y = Q^- / (K * t * A)$$

Y = estimated density of mitochondria in a volume

Q^- = the number of disappearing profiles

(K * t * A) = the estimated total mitochondrial volume

K = the number of sections

t = the section thickness

A = profile area of mitochondria on section #1

$$A = \sum (p) * d^2$$

p = number of grid intersections in mitochondria

d = distance between lines of the grid

1 / Y = estimated mitochondrial volume

Obtaining sequences for cloning

In order to design primers for quantitative real-time PCR (qPCR) in the crucian carp, gene sequences needed to be obtained by cloning and the protocol used was adapted from Fagernes *et al*. (2017). In brief, primer pairs for each gene were designed from zebrafish (*Danio rerio*) sequences (retrieved from the Ensembl database [http://www.ensembl.org/index.html]) using the nucleotide sequence designer program Primer3 (http://primer3.ut.ee; (Rozen and Skaletsky, 2000)). The zebrafish genome was used to design primers because belong to the same family (Cyprinidae) as crucian carp. Sequences were aligned and annotated using GeneDoc (version 2.7; http://www.psc.edu/biomed/genedoc) and ClustalX (version 2.0.12; Chenna *et al*. [2003]). Primer pairs were synthesized by Thermo Scientific (Ulm, Germany).

For cloning, TRIzol (Invitrogen, Carlsbad, CA, USA) and a stand-clamp mounted rotor-stator drill homogenizer (Ultra-Turrax T8, IKA, Staufen, Germany) were used to extract total RNA from normoxic crucian carp tissues. The quantity and quality of extracted total RNA was assessed using a NanoDrop 2000 UV-Vis spectrophotometer (Thermo Fisher Scientific, Rockland, DE, USA). Total RNA samples were stored at -80°C. To reduce risk of any genomic DNA contamination, extracted total RNA was treated with TURBO DNAse (Ambion Applied Biosystems, Foster City, CA, USA). The resultant purified total RNA was used to synthesize cDNA in duplicate with SuperScript III reverse transcriptase (Invitrogen) and oligo(dT)$_{18}$. PCR was carried out using gene-specific primers and cDNA was stored at -20°C. The pGEM®-T Easy Vector System I (Promega, Madison, WI, USA) was used to transform the PCR products into *Escherichia coli* (*E. coli*) cells (TOP10 F; Invitrogen, Carlsbad, CA, USA) that were cultured on LB plates containing ampicillin and IPTG/X-gal (Promega, Madison, WI, USA). PCR was used to verify that positive colonies had inserts of the correct size. Eight colonies of each gene were cleaned with ExoSAP-IT (Affymetrix, Cleveland, OH, USA) and sequenced (GATC, Cologne, Germany) using T7 primers (Invitrogen, Carlsbad, CA, USA). In order to increase the likelihood that paralogs were not being overlooked, several primer pairs were used and multiple products were sequenced for each primer pair (GATC, Cologne, Germany). To confirm the cloned product, the sequences were nucleotide Blasted against the zebrafish and human genome (https://blast.ncbi.nlm.nih.gov/Blast.cgi).

qPCR

Tissue samples from -80°C were homogenized with a stand-clamp mounted rotor-stator drill homogenizer and RNA was extracted in TRIzol (Invitrogen, Carlsbad, CA, USA). An external standard RNA control gene *mw2060* (Ellefsen et al., 2008) was added to each sample on a per-mg-tissue-basis prior to tissue homogenization. Traditionally, qPCR results are presented relative to housekeeping genes, which perform the role of an internal standard. These genes are selected based on the assumption that they are unchanging in response to the treatment applied. A problem posed by acclimation to anoxia is that reference genes are likely affected by differential regulation and a decrease in total RNA (Smith *et al*., 1996; Storey and Storey, 2004; Stecyk *et al*., 2012). For examples of the technique using *mw2060* that addresses this issue, see (Ellefsen et al., 2012; Stecyk et al., 2012). The homogenization of heart and RM were carried out in separate batches and treatments were homogenized randomly within a batch.

To reduce risk of any genomic DNA contamination, extracted total RNA was treated with TURBO DNAse (Ambion Applied Biosystems, Foster City, CA, USA). The resultant purified total RNA was used to synthesize cDNA in duplicate with SuperScript III reverse transcriptase. The crossing point, primer efficiency, and melting peak were determined for each of the primer pairs. Primer specificity was evaluated by melting curve analysis and primer efficiency was estimated with LinReg (Ruijter et

al., 2009). The primer pairs chosen for qPCR were those of the three with the lowest crossing point, highest efficiency, and a single melting peak curve. Additionally, primer pair products were sequenced in order to ensure a single amplicon (GATC, Cologne, Germany). Primer pair for qPCR can be found in supplemental table 3.

The qPCR program used was: 1. 95°C, 10 min; 2. 95°C, 10 sec; 3. 60°C, 10 sec; 4. 72°C, 13 sec; 5. Repeat steps 2. to 4., 42 times. After the qPCR program finished a melting curve analysis was performed for each amplicon. A heat-stable DNA polymerase was used along with a dye (LightCycler 480 SYBR Green I Master Mix; Roche Diagnostics, Basel, Switzerland) that binds specifically to double-stranded DNA. Reaction mixtures and samples were loaded into 384 multiwell plates (Roche Diagnostics) using an Agilent Bravo robot (Agilent Technologies, USA).

For each sample, qPCR was carried out in duplicate on both cDNA syntheses. As the amount of double-stranded cDNA in the reaction mixture increases, so too does the fluorescence intensity. Based on the principle that each cycle the amount of cDNA should double, the Cp can be used to determine the initial number of transcript copies in the sample. In other words, if a low Cp signifies a high amount of mRNA then a sample that reaches its Cp earlier must have more mRNA present in the original sample. The relative mRNA expression levels were calculated using the second derivative maximum method (Roche Lightcycler 480) and the LinRegPCR software (Ruijter et al., 2009) for each reaction well. The Cp was determined based on the threshold value (crossing point), which is the fluorescence value when this threshold is crossed and was obtained from the program. Primer efficiencies were obtained from the raw fluorescence data using the software (Roche) in the LinReg software. Final values are presented relative to a reference gene, which is assumed to be constant between the control and treatment and in this case was the *mw2060* (described above).The following formula was used to calculate relative mRNA expression of a sample:

$$(E_{mw2060}\text{^}Cp_{mw2060})/(E_{tar}\text{^}Cp_{tar})$$

E = mean primer efficiency of the primer pair

Cp = mean crossing point of the two duplicate qPCR reactions

tar = target gene

mw2060 = external standard

Metabolomics

Tissues were sampled as described above and transported on dry ice to the Human Metabolome Technologies (HMT) facility in Yamagata, Japan. The samples arrived with sufficient dry ice remaining so as to guarantee that no thawing occurred. For examples of publications where HMT services was provided see (Makinoshima et al., 2014). Metabolomics was performed on hearts from 6 normoxic and 6 anoxic crucian carp. For specifics of the methods used see (Soga and Heiger, 2000;

Journal of Experimental Biology • Supplementary information

Soga et al., 2003; Soga et al., 2002). In brief, samples were added to 50% acetonitrile in water containing internal standards (20μM for cationic metabolites and 5μM for anionic metabolites). Tissues were then homogenized (1,500 rpm, 120 sec, 3 times) and the supernatant (400 μL x 2) was filtered through a 5-kDa cut-off filter (ULTRAFREE-MC-PLHCC, Human Metabolome Technologies, Yamagata, Japan) in order to remove macromolecules. The filtrate was concentrated by centrifugation and then resuspended in 50 μL of ultrapure water immediately before measurements.

References

Bliksoen, M., Mariero, L. H., Torp, M. K., Baysa, A., Ytrehus, K., Haugen, F., Seljeflot, I., Vaage, J., Valen, G. and Stenslokken, K. O. (2016). Extracellular mtDNA activates NF-kappaB via toll-like receptor 9 and induces cell death in cardiomyocytes. *Basic Res Cardiol* **111**, 42.

Brady, N. R., Elmore, S. P., van Beek, J. J., Krab, K., Courtoy, P. J., Hue, L. and Westerhoff, H. V. (2004). Coordinated behavior of mitochondria in both space and time: a reactive oxygen species-activated wave of mitochondrial depolarization. *Biophys J* **87**, 2022-34.

Ellefsen, S., Bliksoen, M., Rutkovskiy, A., Johansen, I. B., Kaljusto, M. L., Nilsson, G. E., Vaage, J. I. and Stenslokken, K. O. (2012). Per-unit-living tissue normalization of real-time RT-PCR data in ischemic rat hearts. *Physiological genomics* **44**, 651-6.

Ellefsen, S., Stenslokken, K. O., Sandvik, G. K., Kristensen, T. A. and Nilsson, G. E. (2008). Improved normalization of real-time reverse transcriptase polymerase chain reaction data using an external RNA control. *Analytical Biochemistry* **376**, 83-93.

Garcia, Y., Breen, A., Burugapalli, K., Dockery, P. and Pandit, A. (2007). Stereological methods to assess tissue response for tissue-engineered scaffolds. *Biomaterials* **28**, 175-86.

Makinoshima, H., Takita, M., Matsumoto, S., Yagishita, A., Owada, S., Esumi, H. and Tsuchihara, K. (2014). Epidermal growth factor receptor (EGFR) signaling regulates global metabolic pathways in EGFR-mutated lung adenocarcinoma. *The Journal of biological chemistry* **289**, 20813-23.

Mandarim-de-Lacerda, C. A. (2003). Stereological tools in biomedical research. *An Acad Bras Cienc* **75**, 469-86.

Mayhew, T. M. (1992). A review of recent advances in stereology for quantifying neural structure. *J Neurocytol* **21**, 313-28.

Perry, S. W., Norman, J. P., Barbieri, J., Brown, E. B. and Gelbard, H. A. (2011). Mitochondrial membrane potential probes and the proton gradient: a practical usage guide. *Biotechniques* **50**, 98-115.

Rozen, S. and Skaletsky, H. (2000). Primer3 on the WWW for general users and for biologist programmers. *Methods Mol Biol* **132**, 365-86.

Ruijter, J. M., Ramakers, C., Hoogaars, W. M., Karlen, Y., Bakker, O., van den Hoff, M. J. and Moorman, A. F. (2009). Amplification efficiency: linking baseline and bias in the analysis of quantitative PCR data. *Nucleic Acids Research* **37**, e45.

Soga, T. and Heiger, D. N. (2000). Amino acid analysis by capillary electrophoresis electrospray ionization mass spectrometry. *Anal Chem* **72**, 1236-41.

Soga, T., Ohashi, Y., Ueno, Y., Naraoka, H., Tomita, M. and Nishioka, T. (2003). Quantitative metabolome analysis using capillary electrophoresis mass spectrometry. *J Proteome Res* **2**, 488-94.

Soga, T., Ueno, Y., Naraoka, H., Ohashi, Y., Tomita, M. and Nishioka, T. (2002). Simultaneous determination of anionic intermediates for Bacillus subtilis metabolic pathways by capillary electrophoresis electrospray ionization mass spectrometry. *Anal Chem* **74**, 2233-9.

Stecyk, J. A., Couturier, C. S., Fagernes, C. E., Ellefsen, S. and Nilsson, G. E. (2012). Quantification of heat shock protein mRNA expression in warm and cold anoxic turtles (Trachemys scripta) using an external RNA control for normalization. *Comp Biochem Physiol Part D Genomics Proteomics* **7**, 59-72.

West, M. J. (2012). Estimating volume in biological structures. *Cold Spring Harb Protoc* **2012**, 1129-39.

Table S1.

A) mRNA expression of ATP synthase subunits in heart and red muscle

Gene	Portion	Subunit	Treatment			P
			Normoxia	Anoxia 1W	Reoxygenation	
**Heart**						
ATPAF2	F_1	A. fac. alpha	0.010±0.008	0.008±0.006	0.008±0.005	
ATPAF1		A. fac. beta	0.011±0.007	0.008±0.005	0.012±0.006	
ATPF1g		gamma	0.946±0.439	0.242±0.090*	0.411±0.260*	0.001, 0.026
ATPFoCA	F_0	c	0.910±0.421	0.292±0.093*	0.453±0.271*	0.001, 0.030
ATPFoCB			2.526±1.283	0.762±0.234*	1.042±0.596*	0.001, 0.009
ATP5g1a			0.195±0.074	0.044±0.018*	0.081±0.049*	0.001, 0.020
ATP5g1b			0.568±0.237	0.138±0.063*	0.262±0.157	0.001, 0.051
ATP5g3a			0.145±0.058	0.040±0.018*	0.058±0.031*	0.001, 0.010
ATPFoEA1		e	0.166±0.075	0.132±0.054	0.122±0.038	
ATPFoEA2			0.146±0.059	0.108±0.044	0.107±0.036	
ATPFoEB1			0.313±0.076	0.139±0.051*	0.175±0.089*	0.001, 0.001
ATPFoEB2			0.115±0.016	0.070±0.028*	0.077±0.022*	0.001, 0.002
**Red muscle**						
ATPAF2	F_1	A. fac. alpha	0.013±0.009	0.006±0.004	0.009±0.003	
ATPAF1		A. fac. beta	0.009±0.003*	0.006±0.002	0.009±0.002*	0.034, 0.034
ATPF1g		gamma	0.137±0.091	0.121±0.053	0.186±0.048	
ATPFoCA	F_0	c	0.165±0.123	0.138±0.067	0.229±0.059	
ATPFoCB			0.373±0.281	0.306±0.163	0.479±0.136	
ATP5g1a			0.021±0.017	0.024±0.010	0.024±0.010	
ATP5g1b			0.045±0.036	0.036±0.024	0.068±0.029	
ATP5g3a			0.020±0.014	0.012±0.005	0.018±0.007	
ATPFoEA1		e	0.066±0.031	0.042±0.017	0.071±0.017*	0.018
ATPFoEA2			0.061±0.028*	0.038±0.016	0.065±0.016*	0.048, 0.030
ATPFoEB1			0.098±0.056	0.067±0.021	0.101±0.011	
ATPFoEB2			0.053±0.035	0.035±0.017	0.055±0.008	

B): mRNA expression in heart and red muscle

Heart		Treatment			P
Gene	Paralog	Normoxia	Anoxia 1W	Reoxygenation	
VDAC1	a	0.83±0.08	0.84±0.19	0.83±0005	
VDAC1	b	0.40±0.06	0.49±0.05	0.38±0.04	
VDAC2		1.48±0.13	1.27±0.16	1.45±0.15	
VDAC3		0.72±0.11	0.74±.012	0.80±0.17	
HSP60		0.60±0.06	0.45±0.06	0.87±0.14	0.06
Pic 1		0.009±0.005	0.007±0.005	0.003±0.003	
Pic 2		1.23±0.78	0.98±0.73	0.49±0.22	
Cits 2		0.13±0.06	0.08±0.01	0.09±0.04	
Hex1		0.19±0.09a	0.25±0.09a	0.45±0.18b	0.01, 0.03
Hex2.2		0.0008±0.0006a	0.0055±0.001b	0.009±0.004b	0.02, 0.0002
Hex4.1		0.002±0.001a	0.01±0.007b	0.002±0.01b	0.02, 0.0004
Hex4.2		0.0005±0.0003a	0.0008±0.0005ab	0.002±0.0007b	0.001

Heart		Treatment			
		Normoxia	Anoxia 1W	Reoxygenation	P
Gene	Paralog				
Ant1		0.007±0.009	0.0095±0.018	0.0036±0.004	
Ant2.1		12.0±5.8a	6.1±2.9b	8.±5.0ab	0.014
Ant2.2		0.06±0.36a	0.36±0.26b	0.05±0.21a	0.006, 0.03
Ant3.1		7.2E-5±2.7E-6a	1,73E-5±9.7E-6b	1.13E-5±9.33E-6b	0.04, 0.001
Ant3.2		0.0018±0.0008a	0.0012±0.0006b	0.0011±0.0007b	
Cyc D		0.041±0.01a	0.048±0.027a	0.094±0.046b	0.03, 0.03
tFAM1		0.0055±0.004	0.0059±0.008	0.0033±0.001	
tFAM2		0.0036±0.003	0.0013±0.0007	0.0015±0.001	
IfA		0.63±0.15	1.14±1.29	0.89±0.32	
IfB		0.004±0.003	0.03±0.06	0.008±0.005	
Mit1.1		0.089±0.01a	0.16±0.074b	0.14±0.038b	0.03, 0.02
Mit1.2		0.25±0.04	0.23±0.068	0.27±0.07	

Red muscle		Treatment			
		Normoxia	Anoxia 7 days	Reoxygenation	
Gene	Paralog				
VDAC1	a	0.07±0.01	0.08±0.01	0.08±0.01	
VDAC1	b	0.02±0.00	0.02±0.00	0.02±0.00	
VDAC2		2.72±0.47	1.80±0.31	2.04±0.17	
VDAC3		0.56±0.05	0.55±0.05	0.62±0.07	
HSP60		0.27±0.03	0.27±0.03	0.20±0.03	
Pic 1		0.001±0.0008	0.001±0.0006	0.001±0.0004	
Pic 2		0.16±0.11	0.15±0.08	0.21±0.07	
Cits 2		0.04±0.02	0.03±0.02	0.05±0.01	
Hex1		0.02±0.01a	0.05±0.02ab	0.26±0.14b	0.001, 0.02
Hex2.2		0.006±0.003a	0.007±0.003ab	0.010±0.003b	0.04
Hex4.1		8.6E-5±7.4E-5	4.84E-5±3.14E-5	8.83E-5±6.6E-5	
Hex4.2		8.03E-6±7.1E-6	4.7E-6±3.9E-6	8.01E-6±4.32E-6	
Ant1		0.59±0.63	0.22±0.12	0.58±0.41	
Ant2.1		1.50±1.12a	1.00±0.68a	2.53±1.44ab	0.02
Ant2.2		0.04±0.03	0.03±0.01	0.08±0.07	
Ant3.1		1.74E-5±1.58E-5	4E-6±9.28E-7	1.05E-5±1.61E-5	
Ant3.2		0.0003±8.1E-5	0.0002±0.0001	0.0003±7.76E-5	
Cyc D		0.088±0.038a	0.06±0.021ab	0.042±0.015b	0.009
tFAM1		0.002±0.0007a	0.001±0.0002b	0.0029±0.001a	0.009, 0.03
tFAM2		0.0025±0.0016a	0.0012±0.0005b	0.0016±0.00058ab	0.02
IfA		1.15±0.47	0.84±0.32	1.09±0.46	
IfB		0.004±0.002	0.003±0.002	0.005±0.004	
Mit1.1		0.023±0.006	0.023±0.004	0.022±0.006	
Mit1.2		0.036±0.01	0.046±0.01	0.050±0.019	

mRNA expression of genes (left column) in crucian carp heart and skeletal muscle exposed to anoxia (<0.01mg O_2 l^{-1}) for one week (1W, n=8), one week anoxia followed by one week of normoxia (reoxygenation, n=6) compared to normoxic fish (n=6). Values are ± SD. Gene expression data is normalized to external standard (mw2060) A Kruskal-Wallis was used and a Dunn's post-test was

sued if (*p<0.05). Exact p values for the a Dunn's post-test in the right column. An asterix in table A shows difference from control, while different letter in table B shows significant difference between all groups. Voltage dependent anion channel (VDAC, heat shock protein 60 (HSP 60), phosphate carrier (Pic), citrate carrier (Cits), Hexokinase (Hex), Adenine nucleotide translocator (Ant), Cyclophiline D (Cyc D), mitochondrial transcription factor A (tFAM), inhibitor factor 1 (If), Mitofusin 2 (Mit2).

Journal of Experimental Biology • Supplementary information

Table S2. Absolute quantification of heart metabolites

HMT DB	Concentration (nmol/g)						
Compound name	Normoxia		Anoxia		Normoxia vs Anoxia		
	Mean	S.D.	Mean	S.D.	Ratio ¶	*p* -value ‖	
2,3-Diphosphoglyceric acid	0,7	0,08	1,0	0,2	0,7	0,018	*
2-Hydroxyglutaric acid	2,2	1,5	17	5,3	0,13	6,3E-04	***
2-Oxoglutaric acid	18	5,8	N.A.	N.A.	1<	N.A.	
2-Oxoisovaleric acid	N.A.	N.A.	N.A.	N.A.	N.A.	N.A.	
2-Phosphoglyceric acid	0,5	0,4	0,2	N.A.	3,4	N.A.	
3-Phosphoglyceric acid	6,4	3,3	0,7	0,7	9,4	0,007	**
6-Phosphogluconic acid	1,7	0,7	0,7	0,05	2,6	0,012	*
Acetoacetyl CoA	N.A.	N.A.	N.A.	N.A.	N.A.	N.A.	
Acetyl CoA	2,5	1,6	2,7	1,5	0,9	0,830	
Adenine	1,1	0,5	0,5	0,3	2,0	0,055	
Adenosine	123	66	141	95	0,9	0,709	
Adenylate Energy Charge	1,0	0,006	0,8	0,12	1,2	0,036	*
Adenylosuccinic acid	2,0	1,0	1,4	0,5	1,5	0,164	
ADP	42	20	144	64	0,3	0,009	**
ADP-ribose	1,5	0,8	1,1	0,6	1,3	0,379	
Ala	2 246	667	2 871	851	0,8	0,189	
AMP	7,7	4,0	106	75	0,07	0,023	*
Arg	46	22	53	25	0,9	0,616	
Argininosuccinic acid	1,5	0,7	1,1	N.A.	1,4	N.A.	
Asn	7,6	11	15	8,8	0,5	0,393	
Asp	530	362	118	25	4,5	0,038	*
ATP	2 441	705	1 209	675	2,0	0,011	*
Betaine	211	236	227	143	0,9	0,896	
Betaine aldehyde	N.A.	N.A.	N.A.	N.A.	N.A.	N.A.	
cAMP	1,4	0,5	0,8	0,3	1,8	0,031	*
Carbamoylphosphate	N.A.	N.A.	N.A.	N.A.	N.A.	N.A.	
Carnitine	61	40	26	N.A.	2,3	N.A.	
Carnosine	N.A.	N.A.	N.A.	N.A.	N.A.	N.A.	
cGMP	N.A.	N.A.	N.A.	N.A.	N.A.	N.A.	
Choline	81	47	101	39	0,8	0,430	
cis -Aconitic acid	1,5	1,0	0,3	0,2	4,5	0,030	*
Citric acid	189	61	78	57	2,4	0,009	**
Citrulline	18	5,0	29	8,7	0,6	0,020	*
CoA	4,1	2,7	2,7	1,0	1,5	0,285	
Creatine	3 475	865	3 762	469	0,9	0,495	
Creatinine	29	26	34	24	0,8	0,721	
Cys	1,1	N.A.	1,7	N.A.	0,7	N.A.	
Cystathionine	150	121	120	139	1,3	0,694	
Dihydroxyacetone phosphate	50	34	155	65	0,3	0,009	**
Erythrose 4-phosphate	N.A.	N.A.	N.A.	N.A.	N.A.	N.A.	
Folic acid	N.A.	N.A.	N.A.	N.A.	N.A.	N.A.	
Fructose 1,6-diphosphate	27	8,7	94	62	0,3	0,045	*
Fructose 1-phosphate	N.A.	N.A.	121	52	<1	N.A.	
Fructose 6-phosphate	40	11	102	60	0,4	0,052	
Fumarate	22	13	3,6	1,7	5,9	0,032	*
Galactose 1-phosphate	5,1	2,0	2,3	1,0	2,2	0,017	*
GDP	6,9	2,9	8,7	3,9	0,8	0,368	
Gln	760	457	735	229	1,0	0,907	
Glu	2 222	1 145	2 651	655	0,8	0,449	
Glucose 1-phosphate	28	14	15	14	1,9	0,132	

Journal of Experimental Biology • Supplementary information

HMT DB	Concentration (nmol/g)						
Compound name	Normoxia		Anoxia		Normoxia vs Anoxia		
	Mean	S.D.	Mean	S.D.	Ratio ¶	*p*-value ‖	
Glucose 6-phosphate	166	59	93	28	1,8	0,028	*
Glutathione (GSH)	251	113	347	173	0,7	0,287	
Glutathione (GSSG)	206	92	148	94	1,4	0,312	
Gly	1 252	394	695	227	1,8	0,017	*
Glyceraldehyde 3-phosphate	1,0	1,3	11	5,2	0,09	0,004	**
Glycerol 3-phosphate	26	9,7	1 165	153	0,02	8,6E-06	***
Glycerol 3-phosphate/DHAP	0,6	0,3	8,4	3,1	0,08	0,002	**
Glycolic acid	N.A.	N.A.	N.A.	N.A.	N.A.	N.A.	
Glyoxylate	N.A.	N.A.	N.A.	N.A.	N.A.	N.A.	
GMP	6,4	1,8	8,5	2,9	0,7	0,160	
GSH/GSSG	1,6	1,3	5,2	6,7	0,3	0,253	
GTP	370	59	70	20	5,3	1,9E-05	***
Guanine	N.A.	N.A.	0,3	N.A.	<1	N.A.	
Guanosine	10	4,9	5,1	2,1	2,0	0,057	
Guanylate Energy Charge	1,0	0,007	0,8	0,08	1,2	0,012	*
His	588	318	426	225	1,4	0,335	
HMG CoA	N.A.	N.A.	N.A.	N.A.	N.A.	N.A.	
Homocysteine	N.A.	N.A.	N.A.	N.A.	N.A.	N.A.	
Homoserine	N.A.	N.A.	N.A.	N.A.	N.A.	N.A.	
Hydroxyproline	24	14	21	7,3	1,1	0,705	
Hypoxanthine	4,2	2,6	19	12	0,2	0,091	
Isoleucine	72	21	344	83	0,2	3,3E-04	***
IMP	149	195	34	17	4,4	0,209	
Inosine	251	344	33	7,7	7,6	0,182	
Isocitrate	N.A.	N.A.	N.A.	N.A.	N.A.	N.A.	
Lactate/Pyruvate	34	22	605	127	0,06	0,003	**
Lactate	1 735	875	14 791	1 490	0,12	6,7E-08	***
Leucine	153	48	639	151	0,2	2,9E-04	***
Lysine	151	58	1 441	293	0,10	8,2E-05	***
Malate	140	65	48	36	2,9	0,017	*
Malonyl CoA	0,3	0,13	0,2	0,04	1,2	0,484	
Methionine	68	21	74	30	0,9	0,704	
Mevalonic acid	N.A.	N.A.	2,8	1,4	<1	N.A.	
N,*N*-Dimethylglycine	11	3,8	12	2,4	0,9	0,710	
N-Acetylglutamic acid	0,2	0,12	0,5	0,2	0,3	0,052	
NAD^+	195	37	182	9,4	1,1	0,444	
NADH	6,4	0,2	9,6	2,5	0,7	0,027	*
NADH/NAD+	0,03	0,006	0,05	0,013	0,6	0,014	*
$NADP^+$	28	8,2	17	4,7	1,7	0,021	*
NADPH	N.A.	N.A.	4,2	0,13	<1	N.A.	
NADPH/NADP+	N.A.	N.A.	0,3	0,09	<1	N.A.	
N-Carbamoylaspartic acid	0,3	0,3	4,2	6,1	0,07	0,179	
Ornithine	23	14	189	122	0,12	0,020	*
Phenylalanine	55	21	190	50	0,3	5,4E-04	***
Phosphocreatine	479	256	66	74	7,2	0,010	**
Phosphoenolpyruvic acid	N.A.	N.A.	N.A.	N.A.	N.A.	N.A.	
Proline	264	140	560	223	0,5	0,027	*
PRPP	2,2	1,0	0,7	0,5	3,3	0,012	*
Putrescine	100	25	103	31	1,0	0,859	
Pyruvate	53	14	25	4,7	2,1	0,003	**

Journal of Experimental Biology • Supplementary information

HMT DB	Concentration (nmol/g)						
Compound name	Normoxia		Anoxia		Normoxia vs Anoxia		
	Mean	S.D.	Mean	S.D.	Ratio ¶	*p*-value ‖	
Ribose 1-phosphate	4,8	3,2	3,3	1,6	1,5	0,336	
Ribose 5-phosphate	0,8	1,1	N.A.	N.A.	1<	N.A.	
Ribulose 5-phosphate	1,5	1,3	0,9	0,7	1,6	0,465	
S-Adenosylhomocysteine	1,3	0,6	0,7	0,4	1,9	0,170	
S-Adenosylmethionine	7,9	0,8	8,4	2,1	0,9	0,644	
Sarcosine	6,5	2,7	34	14	0,2	0,005	**
Sedoheptulose 7-phosphate	6,0	4,6	N.A.	N.A.	1<	N.A.	
Serine	536	191	488	212	1,1	0,690	
Spermidine	3,1	1,1	5,2	2,6	0,6	0,107	
Spermine	N.A.	N.A.	5,3	N.A.	<1	N.A.	
Succinate	27	10	399	274	0,07	0,021	*
Threonine	104	31	261	113	0,4	0,018	*
Total Adenylate	2 490	708	1 459	590	1,7	0,021	*
Total Glutathione	662	147	644	58	1,0	0,780	
Total Guanylate	383	61	87	15	4,4	4,0E-05	***
Tryptophan	17	2,1	55	13	0,3	6,5E-04	***
Tyrosin	28	11	126	37	0,2	8,3E-04	***
UDP-glucose	25	7,6	9,8	2,9	2,6	0,003	**
Urea	404	282	373	265	1,1	0,874	
Uric acid	1,6	0,5	158	58	0,010	0,001	**
Valine	116	31	511	137	0,2	6,6E-04	***
Xanthine	0,8	0,4	7,8	7,3	0,10	0,064	
XMP	0,4	0,14	0,6	0,4	0,7	0,261	
Xylulose 5-phosphate	N.A.	N.A.	N.A.	N.A.	N.A.	N.A.	
β-Alanine	37	16	19	7,4	2,0	0,040	*
γ-Aminobutyric acid	32	17	19	7,0	1,6	0,144	

HMT DB	Concentration (nmol/g)						
Total compounds per group or ratios	Normoxia		Anoxia		Normoxia vs Anoxia		
	Mean	S.D.	Mean	S.D.	Ratio ¶	*p*-value ‖	
Total Amino Acids	9 168	2 018	12 253	2 784	0,7	0,055	
Total Essential Amino Acids	1 324	385	3 941	779	0,3	1,2E-04	***
Total Non-essential Amino Acids	7 844	2 061	8 312	2 196	0,9	0,711	
Total Glucogenic Amino Acids	8 864	1 958	10 172	2 491	0,9	0,337	
Total Ketogenic Amino Acids	579	121	3 056	690	0,2	2,5E-04	***
Total BCAA	341	98	1 494	369	0,2	3,9E-04	***
Total Aromatic Amino Acids	100	33	371	98	0,3	6,1E-04	***
Fischer's Ratio	3,6	1,2	4,1	0,5	0,9	0,389	
Total Glu-related Amino Acids	3 837	1 380	4 425	1 041	0,9	0,425	
Total Pyr-related Amino Acids	4 155	1 043	4 370	1 351	1,0	0,764	
Total Acetyl CoA-related Amino Acids	393	102	2 479	509	0,2	1,2E-04	***
Total Fumarate-related Amino Acids	82	31	316	86	0,3	6,3E-04	***
Total Succinyl CoA-related Amino Acids	256	68	928	240	0,3	6,7E-04	***
Total Oxaloacetate-related Amino Acids	534	369	132	19	4,0	0,045	*
Malate/Aspartate	0,4	0,3	0,5	0,4	0,7	0,584	
Citrulline/Ornithine	1,0	0,5	0,3	0,3	3,6	0,016	*
Glutamate/2-Oxoglutarate	157	156	N.A.	N.A.	1<	N.A.	
G6P/R5P	429	372	N.A.	N.A.	1<	N.A.	
SAM/SAH	8,6	6,9	14	9,3	0,6	0,432	
Putrescine/Spermidine	36	15	27	21	1,3	0,406	

List of all the compunds analysed by mass spectroscopy in crucian carp hearts eposed to either normoxic (n=6) or one week anoxic hearts (n=6) found in the metabolomic investigation.
Difference in the absolute concentrations of the metabolites was investigated with a Welch corrected t- test. *p<0.05, **p<0.01, ***p<0.001

Journal of Experimental Biology • Supplementary information

Table S3. Accession numbers and primers used

Gene	GenBank ID	Primers for qPCR
mw2060	DQ075244	F- GTGCTGACCATCCGAG<br>R- GCTTGTCCGGTATAACT
ATPAF1	MG099976	F- TCAGACAATGGGAGAAAATGC<br>R- ATCTCGACCAATCGGAAAGTT
ATPAF2	MG099977	F- GGTCTCTGACAGGGCTTGATT<br>R- ATCATGGACCCACTCCACATT
ATPFoCA	MG099978	F- GGCGCTGTACAGACCACTCT<br>R- ACTTATGGCGCTTGTCTGAAA
ATPFoCB	MG099979	F- TGTAGACCACTCTCTGCCTCTG<br>R- GAACCTGCCACTCCCACAGT
ATPFoEA1	MG099980	F- GGTCAGCCTTGCTAATTGGAT<br>R- GGTGTCAGAGTTTGCTTCAGA
ATPFoEA2	MG099981	F- TGATTATCTGAAGCCCATTGC<br>R- CAAAATGGTGTCAGAGTTTGCT
ATPFoEB1	MG099982	F- GTCGCCACTGATTAAGACAGC<br>R- CCTCCTCTTCAATTCTCCTCTC
ATPFoEB2	MG099983	F- GTCTGCATTGCTCGTTGGTAT<br>R- TAGCTGTTTAGCGATGCGTTC
ATPF1g	MG099984	F- GGCAAACACATCCTGATCAAC<br>R- TTTTCTGAGCTTGCAACAGTG
ATP5g1a	MG099985	F- ACCAAAAACAAGAACTGAACAGG<br>R- AATAATGAGGCTGCCGAACA
ATP5g1b	MG099986	F- CTGAGATGAGAACGGAGCAG<br>R- ATAATGAGGCTGCCGAACAC
ATP5g3a	MG099987	F- CTCCAGACCAGCGCTGTAA<br>R- GCCCCATAGCTTCAGACAGA
VDAC1a	MW389890	F- GGCTCCATTTACCAAAAAGTGA<br>R- TAATGTTCCTCCCATCCAGAAG
VDAC1b	MW389891	F- CTTCTCGGCCAAAGTGAATAAT<br>R- CTCAAACTCCAGACCCAAACC
VDAC2	MW389892	F- TATGGATTCGGTATGGTG<br>R- GAAGGTCAGCCCATACTCAGAC
VDAC3	MW389893	F- GGAAACCTGGAGACCAAATACA<br>R- TATCACAGCCCACATTCA
HSP60	MW389894	F- AGATCATTGAGGGCATGAAGTT<br>R- CTGTTAAGGACCAGAGTGCTGA
PiC1	MW389895	F- CAAGTGCTCAAGAAGCTTGGA<br>R- GAGCTTCTTTTTGAGGGATCG
Pic2	MW389896	F- ACAAGTGCTGAAGAAGCTTGGA<br>R- AGCTTCTTTTTGAGGGACTCG
Cits 2	MW389897	F- CTGGCAGACCTCATCCCTAA<br>R-GGCAGCACTGAACTGAGACA
MFN2	MW389898	F- GGCCAAGCAGATTTCTGAAG<br>R- CAGTCTCCGGATCTCCTCTG
IF1-a	MW389899	F- TAAAGGTGCTGGGAAAGGAG<br>R- TTTTGGTGGTGGTCGATTTC
IF-1b	MW389900	F- GAGGAGCGTTTGGGAAGAAG<br>R- GTCGGTCGATCTCTCTCTGC
ANT1	MW389901	F- GGGGTCTCTACCTTGGCTTC<br>R- ACGGTGTCGAAGGGATATGA

Journal of Experimental Biology • Supplementary information

ANT2.1	MW389902	F- TTGAGAGAGTCAAACTGCTGCT<br>R- ATCCTTGAAAGCAAAGTTGAGG
ANT2.2	MW389903	F- GGATATGCAGTACAAGGGCATT<br>R- TGTGCTTGTCTACACCATCGAG
ANT3.1	MW389904	F- GTGTTGATAAGCACACGCAGTT<br>R- TAAAGATTTTGGCCAGACAGTG
ANT3.2	MW389905	F- TTCGTCTACCCTCTGGATTTTG<br>R- GAGGCCCTGTAAATAATGATGC
HEX1	MW389906	F- TCAGGGTGCTTTTGGTAAAGAT<br>R- AAGGAAAAGGTAAAGCCCAGAG
HEX2.2	MW389907	F- TCTCAATCGGGATCAACTCC<br>R- AAGAAGTCGCCATGCTCTGT
HEX4.1	MW389908	F- TCCTGAGCTCACTGGGAATC<br>R-AAGCTTATGGAACCGCTCCT
HEX4.2	MW389909	F- TTTACAACATCCTAAGCACACTGG<br>R- ACAGCGGCGTTCTCTCATTA
Cycl.D	MW389910	F- CAACACCAATGGCTCACAGT<br>R- CAATGCCCCAGTTATCACCT
tFAM1	MW389911	F- CCCAACATCAGCAGACAAAA<br>R- TCTGTGCAGGACTCAGTTGG
tFAM2	MW389912	F- CAGGAGCGCCTTCAATATCT<br>R- TCCTCCCACGATTTGATTTC
MFN1.1	MW389913	F- GTCGTCTCAGCAGGACATGATA<br>R- GCGTAGTCCAGCCTAAAGAGAA
MFN1.2	MW389914	F- CAGACATGCTGAGAATC<br>R- CACAGTTGAGGTCGTAGCTGAG

Partial sequences from cloning of the different crucian carp genes were submitted to Genbank (NCBI; www.ncbi.nlm.nih.gov/). Accession numbers are enlisted here. Primers for qPCR was made against the cloned sequence and are listed in the right column.

Journal of Experimental Biology • Supplementary information
